# An eco-friendly first and second derivative synchronous spectrofluorimetry for quantification of florfenicol in presence of its different degradation products. Application to kinetic stability study

**DOI:** 10.1098/rsos.231642

**Published:** 2024-07-03

**Authors:** Aya Ayman, M. E. K. Wahba, Alaa El-din M. A. El-Gindy, Yasser El-Shabrawy, Aziza E. Mostafa

**Affiliations:** ^1^ Department of Pharmaceutical Analytical Chemistry, Faculty of Pharmacy, Suez Canal University, Ismailia, Egypt; ^2^ Pharmaceutical Chemistry Department, Faculty of Pharmacy, Delta University for Science and Technology, Gamasa 35712, Egypt; ^3^ Department of Pharmaceutical Analytical Chemistry, Faculty of Pharmacy, Mansoura University, Mansoura 35516, Egypt

**Keywords:** florfenicol, synchronous fluorescence spectroscopy, degradation kinetics, greenness

## Abstract

Two rapid, simple, sensitive and selective derivative spectrofluorimetric methods (first and second derivative synchronous spectrofluorimetric (FDSFS and SDSFS) procedures) have been developed for the analysis of florfenicol in the presence of its various degradation products. FDSFS was applied to assay the drug in the presence of its alkaline, oxidative and photolytic degradation products while SDSFS was used to quantify it in the presence of its acidic degradation product. These methods permitted quantification of florfenicol at corresponding *λ*_Em_ of 288, 287, 279 and 284 nm without interferences from any of its degradation products. Full validation procedures were applied to the suggested method according to International Conference of Harmonization guidelines. Moreover, different degradation kinetic parameters were calculated such as half-life (*t*_1/2_), degradation rate constant (*K*) and activation energy (*E*_a_). Using the analytical eco-scale, green analytical procedure index and analytical greenness metric approach AGREE as greenness assessment tools, the proposed method was found to be environmentally friendly.

## 1. Introduction

Florfenicol (FLR) is a thiamphenicol derivative with a wide antibacterial activity [1] since it attaches to the 50S ribosomal subunit of bacteria, inhibiting protein synthesis at the peptidyl-transferase stage and showing high efficacy against numerous bacteria that are harmful to aquatic life [2]. It is also used to treat dermatological diseases of bacterial aetiology in dogs and cats, such as otitis [3]. Furthermore, FLR is effective against digestive and respiratory tract infections by blocking the growth of *Streptococcus agalactiae* in domestic rabbits [4]. It is chemically designated as 2,2-dichloro-*N*-[(1*R*,2*S*)-3-fluoro-1-hydroxy-1-(4-methanesulfonylphenyl)propan-2-yl] acetamide [5] (electronic supplementary material, figure S1).

Previous studies demonstrated different methods for quantification of FLR either alone or in combination with other drugs, including spectrophotometry [6–8], high-performance liquid chromatography (HPLC) [9–16], voltammetry [17] and gas chromatography (GC) [18,19].

Owing to the inappropriate use of FLR in clinical veterinary medicine, substantial resistance issues have arisen, as well as the building up of residues in animal products, in addition to potential anaphylactic reactions that they may be prohibitive in some individuals [20]. In terms of both qualitative and quantitative analysis, it is extremely important to conduct a detailed quality control study for veterinary drugs. Stability study is an integral part of any drug development regarding the quality of drug product changes over time under the effect of different factors such as light, temperature, pH of solvent and humidity [21].

The goal of a stability-indicating test is to discover the changes that occur during storage that are likely to affect the quality, safety, efficacy, shelf-life and hence, the recommended storage conditions [21].

Forced stability studies of drug substances represent a useful guide to identify the produced degradation products and hence to display the intrinsic stability of the compound, in addition to establishing the possible degradation pathways. Consequently, the process of confirming that the proposed method can accurately determine the stability of a compound by differentiating it from its degradants is of utmost importance and necessitates its subjection to a detailed validation study. This validation involves ensuring that the method meets specific criteria such as high accuracy and precision, specificity, low detection and quantification limits, acceptable linearity and wide range. Stress testing should include the effect of many variables like the influence of heat, oxidation, photolysis and susceptibility to hydrolysis over a wide pH range [21].

Direct fluorescence spectroscopy is a highly sensitive, rapid and nondestructive technique that requires minimal sample pretreatment. For this reason, it is widely used in quality control laboratories. However, single-wavelength spectrofluorimetry has a limited ability to analyse drug mixtures especially when their spectra overlap [22]. Synchronous fluorescence spectroscopy (SFS) overcomes this limitation by narrowing spectral bands, enhancing selectivity and reducing the overlap between the resultant spectra [23]. There are different modes of SFS techniques such as constant wavelength, constant energy and variable angle synchronous luminescence. Currently, the constant wavelength mode is used most frequently, where it involves simultaneous scanning of both excitation and emission wavelengths at constant ∆*λ* values [23].

In terms of sensitivity and selectivity, synchronous fluorimetry combined with derivatization is valuable, often providing high tolerance limits and recovery percentages at concentration levels as low as ng ml^−1^ for organic molecules, metallic species, pharmaceutical compounds and biological samples [23].

Recently, derivative synchronous spectrofluorimetry (DSFS) has shown results that are more consistent with those obtained from HPLC, GC and infrared spectroscopy [23]. Besides, DSFS has been extensively used for quantification of various mixtures like metolazone and valsartan [24], alfuzosin hydrochloride and solifenacin succinate [25], meropenem and ertapenem [26]. This method has also been used for estimating gliquidone concentration in the presence of its alkaline degradation product [27].

The current work is devoted to carrying out a detailed kinetic stability study for FLR under the guidance of the International Conference of Harmonization (ICH) recommendation [21]. Derivative synchronous fluorimetry was applied as an analysis tool, enabling quantification of the analyte under study in the presence of its different degradation products.

## 2. Experimental

### 2.1. Instrument

Shimadzu 6000 RF Spectro fluorophotometer of serial number A402458, supplied with a 150 W xenon lamp (Shimadzu Corporation, Kyoto, Japan).

### 2.2. Reagent and chemicals

FLR of purity 99.9% was kindly supplied from Pharma Swede-Egypt, 10th of Ramadan City, El-Sharqia Governorate, Egypt. Floromed^®^ 30% (solution for injection), a product from Arab company for medical products 6 October, Giza, Egypt, was obtained from a local company (batch no. 0921). Ethanol (HPLC grade) was imported from Thermo Scientific™, Basingstoke, UK. Sodium hydroxide (NaOH; 0.1–1 M, of purity 97%) and hydrochloric acid (HCl; product of Fisher Scientific, UK) were freshly prepared in deionized water. Hydrogen peroxide (H_2_O_2_; 0.07% (v/v) solution) was prepared from 6% stock solution (laboratory grade, USA) by transferring 1.16 ml into a 100.0 ml volumetric flask using deionized water as a diluting solvent.

### 2.3. Standard stock solution

A stock solution of FLR was prepared as 1.0 mg ml^−1^ by dissolving 100.0 mg of FLR in ethanol–deionized water (40 : 60 v/v) using a 100.0 ml volumetric flask. This solution was stable for at least one week when kept in a refrigerator.

## 3. General procedure

### 3.1. Construction of calibration curve

To a series of 10.0 ml volumetric flasks, aliquots of standard solution of FLR over the concentration range 0.5–16.0 µg ml^−1^ were transferred and completed to the mark with a mixture of ethanol and deionized water in a ratio of 40 : 60 (v/v) and mixed well. Synchronous fluorescence spectra were recorded by scanning monochromators at (Δλ) 60 nm. A scan rate of 6000 nm min^–1^ was used, adjusting the bandwidth of the excitation and emission monochromators at 5 nm. For each measurement, a blank experiment was performed concurrently. The resulting SFS spectra were then derivatized using Shimadzu Lab Solution RF^®^ software, where FLR was quantified at 285 nm. The calibration graph (electronic supplementary material, figure S2) was constructed by plotting the corrected peak amplitudes versus the final drug concentration (µg ml^–1^); alternatively, the relevant regression equation was concluded.

### 3.2. Stress testing

The stability study for all types of degradation products was conducted for a final concentration of FLR of 10.0 µg ml^–1^.

#### 3.2.1. Alkaline and acidic degradation

The appropriate volume of FLR stock solution (1.0 mg ml^–1^) was accurately transferred into a series of test tubes then 2.0 ml of either sodium hydroxide or hydrochloric acid of different molarities (0.1, 0.3, 0.5, 0.8 and 1 M) was added. The solutions were kept in a thermostatically controlled water bath of different temperature settings (40°C, 60°C, 80°C and 100°C). The resultant hydrolysed FLR solutions were removed from the water bath at 5-min intervals (for the first 30 min of hydrolysis), and then the rest of the solutions were withdrawn at 15-min time intervals (for the next 60 min of hydrolysis). The hydrolysed solutions were then transferred quantitively to 10.0 ml volumetric flasks and diluted to the mark with a mixture of ethanol and deionized water (40 : 60 v/v). Synchronous fluorescence spectra of the degraded FLR solutions were scanned at different ∆*λ* for both degradation pathways, and then first and second derivative synchronous fluorescence spectra (FDSFS and SDSFS) for alkaline and acidic degradation were derived, respectively. Log *a*/(*a* − *x*) versus time (min) was plotted to get the reaction rate constant and half-life (*t*_1/2_).

#### 3.2.2. Oxidative degradation

To an appropriate volume of FLR stock solution, 2 ml of 0.07% (v/v) H_2_O_2_ was added to a series of test tubes and the solutions were heated at different temperature settings in a thermostatically controlled water bath (25°C, 40°C, 60°C, 80°C and 100°C). The degraded solutions were withdrawn at 5 min intervals (until 30 min), then the steps mentioned in §3.2.1 were followed, where the SFS of the solutions were scanned at ∆*λ* (60 nm), followed by the first derivatization.

#### 3.2.3. Photolytic degradation

The standard solution of FLR of final concentration 10.0 µg ml^−1^ was placed in a wooden cabinet supplied with a UV lamp of 254 nm placed at a distance of 15 cm. Solutions were subjected to UV degradation for different time spans (1–23 h). The SFS of photolytic solutions were then measured at ∆*λ* = 60 nm and the resultant spectra were subjected to first derivatization.

### 3.3. Procedure for ‘solution for injection’

To acquire the working concentration range as mentioned in §3.1, 0.33 ml of Floromed^®^ solution for injection was transferred into a 100.0 ml volumetric flask and diluted with the same solvent ratio to attain stock concentration equivalent to 1.0 mg ml^−1^ of FLR. By using the corresponding regression equation, the nominal content of the injectable solution was calculated.

## 4. Results and discussion

A kinetic stability-indicating study evaluates how the quality of a drug substance or final drug products changes over time as a result of various external environmental factors [28]. This testing determines if any physical, chemical or microbial changes impact the efficacy and safety of pharmaceuticals [28]. This study calculates half-life (*t*_1/2_) and degradation rate constant (*K*) according to ICH guidelines [21] using 10.0 µg ml^−1^ of the studied drug for all degradation pathways.

### 4.1. Acidic and alkaline degradation kinetics

Different molar concentrations of either NaOH or HCl were used (0.1, 0.3, 0.5, 0.8 and 1 M) to conduct the forced stability study applying different temperature settings (ranging from 40°C to 100°C). It was noticed that the SFS of FLR decreased dramatically with a corresponding increase in the concentration of NaOH or HCl. Moreover, the decrease in SFS responses was temperature-dependent.

To quantify FLR in its mixture with either alkaline or acidic degradation products, ∆*λ* selection should be carefully considered, as it plays a vital role in resolution efficiency, sensitivity and specificity. Consequently, different ∆*λ* were applied (20–120 nm) to scan the SFS of FLR whether alone (electronic supplementary material, figure S3*a*) or in the presence of its alkaline or acidic degradation products (electronic supplementary material, figures S3*b*,*c*, S4 and S5), selecting 1 M NaOH and 1 M HCl as model examples, after boiling in a water bath for 45 min. By reviewing the resultant spectra, it is obvious that ∆*λ* = 60 nm was optimum, so it was used during this study. It resulted in better resolution accompanied by symmetric spectra with higher sensitivity measures when compared to other ∆*λ* values for either degradation pathway (electronic supplementary material, figure S3*b*,*c*). Owing to the significant overlap between SFS of FLR in the presence of its degradation products, first derivatization was applied which permitted quantification of FLR at 288 nm in the presence of its alkaline degradation product while second derivatization was performed to determine FLR in the presence of its acidic degradation product, quantifying it at 284 nm (electronic supplementary material, figure S6). Such wavelengths represent the zero-crossing point for either degradation product.

**Figure 1.**
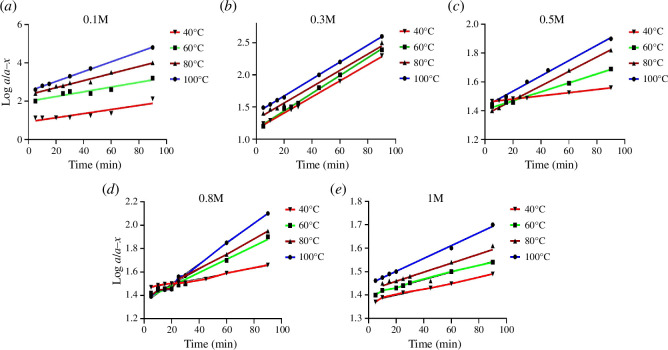
Semilogarithmic plots of FLR (10.0 µg ml^−1^) versus different heating times after heating at different temperature settings with different concentrations of NaOH: (*a*) 0.1 M, (*b*) 0.3 M, (*c*) 0.5 M, (*d*) 0.8 M and (*e*) 1.0 M.

**Figure 2.**
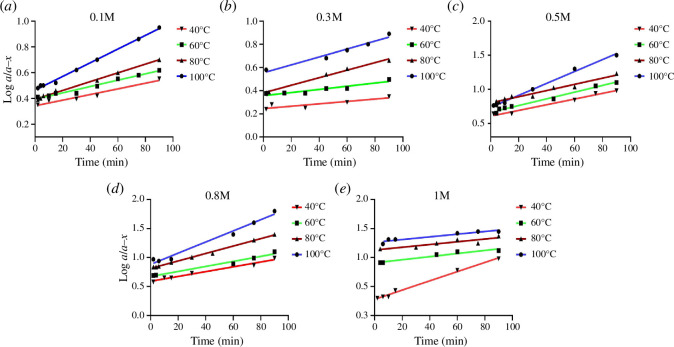
Semilogarithmic plots of FLR (10.0 µg ml^−1^) versus different heating times after heating at different temperature settings with different concentrations of HCl: (*a*) 0.1 M, (*b*) 0.3 M, (*c*) 0.5 M, (*d*) 0.8 M and (*e*) 1.0 M.

To deduce the reaction rate order, the graphical method was applied by plotting log *a*/(*a *− *x*) versus time [29], where *a* is the initial concentration of the drug and (*a* − *x*) is the remaining concentration of FLR after degradation (figures 1 and 2). It is clearly demonstrated from the straight lines obtained that the degradation of FLR follows first-order kinetics.

From the obtained data, it was possible to calculate the reaction rate constant (*K*) and the half-life time (*t*_1/2_) for both alkaline and acidic degradation upon using different molar concentrations of NaOH and HCl of different temperature settings. The obtained results are summarized in table 1. To follow the effect of temperature on the reaction rate constant, Arrhenius plots were derived through plotting log *K* versus 1/*T* (electronic supplementary material, figures S7 and S8), which permitted the determination of the activation energy (*E*_a_) (table 1). It is to be considered that FLR is more liable to alkaline than acidic degradation as revealed from the calculated values of *E*_a_ (*E*_a_ of alkaline egradation < *E*_a_ of acidic degradation at different temperature settings), where it undergoes hydrolysis by 30% and 10%, respectively, upon boiling with 1 M of NaOH or HCl for 45 min.

**Table 1.** Kinetic parameters for alkaline and acidic degradation of FLR using the proposed method. Bold text indicates the studied parameters and the obtained results.

temperature (°C)	alkaline degradation											
	0.1 M			0.3 M			**0.5 M**		0.8 M		1 M	
	*K* (min^−1^)		*t*_1/2_ (h)	*K* (min^−1^)		*t*_1/2_ (h)	*K* (min^−1^)	*t*_1/2_ (h)	*K* (min^−1^)	*t*_1/2_ (h)	*K* (min^−1^)	*t*_1/2_ (h)
40	1.0 × 10^–2^		1.15	1.30 × 10^–2^		0.88	2.1 × 10^–3^	5.5	4.0 × 10^–3^	2.88	1.3 × 10^–3^	8.88
60	1.24 × 10^–2^		0.93	1.38 × 10^–2^		0.83	3.1 × 10^–3^	3.72	6.0 × 10^–3^	1.92	1.6 × 10^–3^	7.21
80	1.84 ×10^–2^		0.62	1.30 × 10^–2^		0.88	5.0 × 10^–3^	2.31	6.5 × 10^–3^	1.77	1.90 × 10^–3^	6.07
100	2.56 × 10^–2^		0.45	1.40 × 10^–2^		0.82	5.3 × 10^–3^	2.17	8.5 × 10^–3^	1.35	2.7 × 10^–3^	4.27
***E*_a_ (kJ mol^−1^)**	**10.14**			**0.76**			**13.40**		**14.16**		**13.59**	
	**acidic degradation**											
	**0.1 M**			**0.3 M**			**0.5 M**		**0.8 M**		**1 M**	
	***K*** **(min^−1^)**	***t*_1/2_ (h)**		***K* (min)^−1^**	***t*_1/2_ (h)**		***K* (min^−1^)**	***t*_1/2_ (h)**	***K* (min^−1^)**	***t*_1/2_ (h)**	***K* (min^−1^)**	***t*_1/2_ (h)**
40	2.19 × 10^–3^	5.27		1.03 × 10^–3^	11.21		4.0 × 10^–3^	2.88	4.2 × 10^–3^	2.75	8.0 × 10^–3^	1.44
60	2.4 × 10^–3^	4.81		1.36 × 10^–3^	8.49		4.9 × 10^–3^	2.35	4.36 × 10^–3^	2.64	2.70 × 10^–3^	4.27
80	3.41 × 10^–3^	3.38		3.25 × 10^–3^	3.55		4.5 × 10^–3^	2.56	6.40 × 10^–3^	1.80	2.40 × 10^–3^	4.81
100	5.3 × 10^–3^	2.17		3.41 × 10^–3^	3.38		8.7 × 10^–3^	1.32	9.62 × 10^–3^	1.20	2.30 × 10^–3^	5.02
***E*_a_ (kJ mol^−1^**)	**17.04**			**25.84**			**14.16**		**14.34**		**15.12**	

### 4.2. Oxidative degradation kinetics

Similarly speaking, SFS was used to assay FLR in a mixture with its oxidative degradation product at ∆ λ = 60 nm which was selected for the same reason as mentioned in the previous section. The overlap in the obtained spectra (electronic supplementary material, figure S9*a*) necessitates conduction of first derivatization (electronic supplementary material, figure S9*b*) which enables determination of FLR at 287 nm (zero-crossing point for the oxidative degradation product).

Using the same concept as mentioned in §3.2.1, the oxidative degradation was found to follow first-order kinetics (electronic supplementary material, figure S10), where the Arrhenius plot and different oxidative kinetic parameters are demonstrated in electronic supplementary material, figure S11, and table 2, respectively.

**Table 2.** Kinetic parameters of the oxidative degradation of FLR using 0.07% (v/v) H_2_O_2_.

temperature (°C)	*K* (min^−1^)	*t*_1/2_ (h)
25	0.012	0.95
40	0.013	0.88
60	0.05	0.39
80	0.06	0.19
100	0.075	0.15
*E*_a_ (J mol^−1^)	**23.55**	

### 4.3. UV photolysis

Photodegradation of 10.0 µg ml^−1^ of FLR at ambient temperature resulted in 38.03% degradation after 23 h of exposure to a 254 nm UV lamp. Due to the overlap of SFS of FLR with that of its photodegradation product at ∆*λ* = 60 nm (electronic supplementary material, figure S12*a*), FDSFS was applied to quantify the concerned analyte at 279 nm (electronic supplementary material, figure S12*b*).

### 4.4. Degradation pathways and antibacterial activity

The release of FLR or either of its degradation products to ground or surface water is crucial for human, livestock and ecosystem safety [30,31].

Pathways of alkaline and acidic degradation of FLR have been investigated before [32], where the degradation products were separated and identified by liquid chromatography–mass spectrometry [32]. By referring to electronic supplementary material, scheme S1, it is obvious that hydrolysis of the amide linkage is the main route of both alkaline and acidic degradation [33], which is followed by defluorination in alkaline degradation producing the hydroxyl derivative instead [32]. Since the resultant degradation products are more polar than the parent drug [30], it is expected that their prevalence in surface water is significant.

The biological activity of FLR is related to the presence of the methylsulfonyl moiety, the fluorine atom, the dichloroacetyl group and the alcoholic group at the third carbon of the propanediol chain [34]. By reviewing the structure of the resultant degraded products, it could be realized that both products are deficient of the dichloroacetyl group, and hence they are expected to have lower antibacterial activity than FLR, keeping in mind that the defluorinated alkaline degradation product is less active than the acidic one. Regarding oxidative degradation, hydroxylation is most likely to occur as documented in a previous report [35] which applied UPLC/DAD as an analysis tool.

Concerning photolysis, defluorination is expected to predominate as formerly studied utilizing UPLC/MS for elucidation of the chemical structure [36]. The resultant oxidative and photolytic products (electronic supplementary material, scheme S1) have almost the structural features required to impact the antibacterial activity as FLR. This implies that their presence in surface water may lead to an enhanced resistance to treatment with FLR, keeping in mind that these two degradation pathways are more likely to take place for FLR environmental residues.

### 4.5. Method validation

Following the ICH guidelines [37], the validity of the suggested method was investigated in terms of linearity, quantitation limit (LOQ), detection limit (LOD), accuracy, precision and specificity.

FLR responses followed a linear pattern over the concentration range of 0.5–16.0 µg ml^−1^ in the presence of any type of its degradation products. Calibration curves were constructed by plotting the peak amplitudes versus the final concentration of the studied drug in µg ml^−1^, which enabled the development of partial last square regression equations (table 3).

**Table 3.** Performance parameters of FLR in the presence of different types of degradation products.

	alkaline	acidic	oxidative	UV
	FDSFS	SDSFS	FDSFS	FDSFS
linearity range (µg ml ^−1^)	0.5–16.0			
regression equations	*Y* = 2217.1*x* + 4952.9	*Y* = 406.4*x* + 1459.5	*Y* = 2090.5*x* + 4664.4	*Y* = 1855.6*x* + 3548.5
limit of detection, LOD (µg ml ^−1^)	0.13	0.15	0.13	0.12
limit of quantification, LOQ (µg ml ^−1^)	0.40	0.45	0.40	0.39
correlation coefficient (*r*)	0.9999	0.9998	0.9999	0.9998
standard deviation of intercept (*S*_a_)	89.33	17.85	84.55	72.55
standard deviation of slope (*S*_b_)	15.46	3.09	14.63	12.55
standard deviation of residual (*S*_*y*/*x*_)	209.12	41.79	197.93	169.83
linearity and range of FLR using the suggested method				
analyte taken (µg ml^−1^)	found (µg ml^−1^)	found (µg ml^−1^)	found (µg ml^−1^)	found (µg ml^−1^)
0.5	0.48	0.49	0.51	0.50
2.0	2.02	1.99	2.03	1.98
4.0	3.95	3.98	3.99	3.95
6.0	6.10	5.99	5.88	6.10
12.0	11.97	12.15	11.94	11.91
16.0	15.98	15.88	16.08	16.04

The linearity of the measured responses with concentration is confirmed by low scattering of the data points around the calibration line, which is corroborated by the correlation coefficient values that approach unity. Furthermore, the validity of the proposed method was evaluated by the regression data regarding standard deviation of residuals (*S*_*y*/*x*_), standard deviation of slope (*S*_b_) and standard deviation of the intercept (S_a_) (table 3).

LOQ is defined in accordance with ICH guidelines [37] as the lowest amount of the analyte that can be quantified. On the other hand, LOD is described as the lowest amount of the analyte that can be detected. The values obtained re listed in table 3, reflecting the sensitivity of the proposed method.

Precision was assessed by analysing three concentrations of FLR on the same day (intraday) or on three successive days (interday). The obtained small relative standard deviation values indicate that the suggested method is precise (table 4). The specificity of the proposed method was revealed by analysis of the interference that could exist from dosage form excipients. High values of percentage recoveries reflected no encountered interference (table 5).

**Table 4.** Precision data for determination FLR in its pure form.

taken (µg ml^–1^)	intraday		interday	
	found (µg ml^–1^)	recovery (%)	found (µg ml^–1^)	recovery (%)
4.0	3.96	97.67	3.89	97.31
6.0	5.97	99.58	5.95	99.30
12.0	11.96	99.73	11.95	99.60
mean ± s.d.		98.99 ± 1.15		98.74 ± 1.24

**Table 5.** Application of the proposed and comparison methods for determination FLR in its injectable solution.

	taken (ml^–1^)	found (ml^–1^)	recovery (%)	comparison method [6]: recovery (%)
Floromed^®^ 30%	2	1.94	97.47	100.44
	4	3.97	99.34	99.1
	6	5.98	99.77	100.44
	10	9.99	99.95	
	12	11.76	98.02	
	14	14.18	101.3	
mean ± s.d.			99.31 ± 1.38	99.99 ± 0.77
*t*-test				0.61 (4.303)^a^
*F*-test				3.87 (19.30)^a^

^a^ Values in parentheses are the tabulated *t-* and *F*-values, at *p* = 0.05 [38].

### 4.6. Application to dosage form

Determination of FLR in its injectable solution was performed using the proposed method. The results obtained were statistically compared with those acquired from the comparison method [6]. Regarding Student *t*-test and the variance ratio *F*-test values [38], there was no significant difference found between the proposed and comparison methods (table 5).

### 4.7. Greenness assessment of the proposed method

Concerns about the negative impact of analytical research on the environment have arisen [39]. Utility of hazardous solvents/chemicals, production of large amounts of untreated waste and carrying out several extraction procedures that may impact health, safety and occupational concerns are the main factors that influence the greenness of an analytical method [39]. To introduce an environmentally benign tool, three reported techniques were applied to evaluate the greenness of the proposed method, namely the analytical eco-scale [40], green analytical procedure index (GAPI) [41] and analytical greenness metric approach (AGREE) [42].

#### 4.7.1. Analytical eco-scale

This tool is based on calculating penalty points (PPs) subtracted from the ideal value of 100. The higher the score, the greener and more cost effective the proposed method [40]. The outcome is evaluated on a scale: >75 indicates excellent green analysis, >50 indicates good green analysis and 50 indicates insufficient green analysis [40]. The suggested method was found to be excellent green with total PPs of 93 (table 6).

**Table 6.** PPs of the suggested method using the analytical eco-scale [40].

parameters	description	PP
**reagents**		
ethanol	amount = 2 ml hazard = 2	PP = 2 × 2 = 4
water	amount = 2 ml hazard = 0	PP = 2 × 0 = 0
**instrument**		
spectrofluorometer	<0.1 kWh per sample	PP = 0
occupational hazard	emission of vapours and gases to the air	PP = 0
waste	1–10 ml	3
total		∑7
total PPs		93

#### 4.7.2. Green analytical procedure index assessment

GAPI is a novel semi-quantitative assessment tool for evaluating the green properties and environmental effects of each stage of analytical methods. The visual depiction of the GAPI provides the researcher with an instantly apparent perspective from which to make their own conclusion concerning competing green criteria [41]. Furthermore, it provides detailed information on the involved procedures by providing a more detailed evaluation for each applied step in the analytical practice by including 15 parameters to be investigated via three levels of colour scale: green, yellow or red, indicating high, medium or low impact, respectively [41].

These examined characteristics are represented by pentagrams. The suggested approach uses harmless solvents and does not involve any pre-extraction operations or specific preparation conditions, resulting in lower energy and producing limited waste. The steps involved in GAPI for evaluating the proposed method are listed in table 7 and the resultant pictograms are presented in electronic supplementary material, figure S13.

**Table 7.** Assessment of the proposed SFS technique using GAPI [41].

category	comment	colour
**sample preparation**		
collection	at line	yellow
preservation	none	green
transport	none	green
storage	none	green
type of method direct/Indirect	direct	green
extraction	none	green
solvents/reagents used	water/ethanol	yellow
additional treatment	none	green
**reagents and solvents**		
amount	10–100 ml	yellow
health hazard	ethanol (2); water (0)	yellow
safety hazard	ethanol (3); water (0)	yellow
**instrumentation**		
energy	<0.1 kWh per sample	green
occupational hazard	hermetic sealing	green
waste	1–10 ml	yellow
waste treatment	biodegradable	yellow

#### 4.7.3. AGREE

For determining greenness, AGREE software employs 12 criteria (electronic supplementary material, figure S14). AGREE: The Analytical Greenness Calculator (v.0.5, Gdansk University of Technology, Gdansk, Poland, 2020) [42] was used to assess the greenness of the proposed method. Electronic supplementary material, figure S14, represents the AGREE pictogram of the promising method and the value was calculated to be 0.77, indicating the greenness of the suggested method.

## 5. Conclusion

A forced stability testing was developed to assay FLR in the presence of its degradation products by applying SFS followed by derivatization. The reaction rate order for acidic, alkaline, oxidative and photolytic degradation of FLR proved to be first order applying the graphical method. Moreover, FLR degradation was found to be temperature-dependent; hence, different kinetic parameters were concluded. The greenness of the suggested method was assessed by three different tools and found to be green.

## Data Availability

Data are uploaded on the Dryad repository system under the title: ‘Synchronous fluorescence spectra for stress testing of florfenicol’ [43]. Electronic supplementary material is available online [44].
